# Carbonic anhydrase XII is a new therapeutic target to overcome chemoresistance in cancer cells

**DOI:** 10.18632/oncotarget.2882

**Published:** 2015-02-18

**Authors:** Joanna Kopecka, Ivana Campia, Andrea Jacobs, Andreas P. Frei, Dario Ghigo, Bernd Wollscheid, Chiara Riganti

**Affiliations:** ^1^ Department of Oncology, University of Torino, 10126 Torino, Italy; ^2^ Department of Biology, Institute of Molecular Systems Biology, Swiss Federal Institute of Technology (ETH) Zurich, 8093 Zurich, Switzerland; ^3^ Biomedical Proteomics Platform (BMPP), Department of Health Sciences and Technology, Swiss Federal Institute of Technology (ETH) Zurich, 8093 Zurich, Switzerland

**Keywords:** chemoresistance, surfaceome, P-glycoprotein, carbonic anhydrase type XII

## Abstract

Multidrug resistance (MDR) in cancer cells is a challenging phenomenon often associated with P-glycoprotein (Pgp) surface expression. Finding new ways to bypass Pgp-mediated MDR still remains a daunting challenge towards the successful treatment of malignant neoplasms such as colorectal cancer.

We applied the Cell Surface Capture technology to chemosensitive and chemoresistant human colon cancer to explore the cell surface proteome of Pgp-expressing cells in a discovery-driven fashion. Comparative quantitative analysis of identified cell surface glycoproteins revealed carbonic anhydrase type XII (CAXII) to be up-regulated on the surface of chemoresistant cells, similarly to Pgp. In cellular models showing an acquired MDR phenotype due to the selective pressure of chemotherapy, the progressive increase of the transcription factor hypoxia-inducible factor-1 alpha was paralleled by the simultaneous up-regulation of Pgp and CAXII. CAXII and Pgp physically interacted at the cell surface. CAXII silencing or pharmacological inhibition with acetazolamide decreased the ATPase activity of Pgp by altering the optimal pH at which Pgp operated and promoted chemosensitization to Pgp substrates in MDR cells.

We propose CAXII as a new secondary marker of the MDR phenotype that influences Pgp activity directly and can be used as a pharmacological target for MDR research and potential treatment.

## INTRODUCTION

One of the main features of chemoresistant cancer cells is the high cell surface expression of ATP binding cassette (ABC) transporters, such as P-glycoprotein (Pgp/ABCB1), multidrug resistance (MDR) related proteins (MRPs/ABCCs) and breast cancer resistance protein (BCRP/ABCG2). These plasma membrane transporters enable the active transport of chemotherapeutic drugs into the extracellular space, ultimately reducing intracellular concentrations, cytotoxicity, and therapeutic success [1–3]. Pgp is one of the ABC transporters with the broadest spectrum of substrates, which include anthracyclines, taxanes, Vinca alkaloids, epipodophyllotoxins, topotecan, methotrexate, imatinib, dasatinib, lapatinib, gefitinib, sorafenib, and erlotinib. As a consequence, tumors overexpressing Pgp often exhibit a MDR phenotype and are difficult to eradicate by chemotherapy [1, 4].

In attempts to overcome chemoresistance, ABC transporters have been targeted with pharmacological inhibitors. However, such approaches have led to several therapeutic failures due to the widespread tissue distribution of ABC transporters. Moreover, since ABC transporters play critical functions in the physiological clearance of catabolites and xenobiotics [5–7], their pharmacological inhibition produces toxicities *in vivo*. Thus, pharmacological targeting of other surface proteins that are selectively overexpressed in chemoresistant cells and modulate the activity of ABC transporters is a promising alternative approach.

Until now, only few studies have analyzed the surfaceome of chemoresistant cells [8, 9]. These studies have identified proteins that were overexpressed in MDR cells, such as CD44 [8], dihydropyridine receptor alpha 2 and laminin subunit alpha 5 [9]. These cell surface proteins could be of clinical utility as potential biomarkers predictive of chemoresistance and/or potential therapeutic targets. However, these proteins were not found to modulate the expression or activity of Pgp or other ABC transporters in MDR cells, making them unlikely targets for the suppression of the MDR phenotype.

Pgp activity is finely modulated by the plasma-membrane lipid composition and physicochemical parameters [10]. A considerable number of proteins have been reported to physically interact with Pgp, such as the E3 ubiquitin ligases RNF2 [11] and FBXO15 [12], the endoplasmic reticulum-associated chaperon calreticulin [13], the serine/threonine kinase Pim-1 [14], the estrogen receptor repressor prohibitin 2 [15], the transcription factor Myc [16], the surface molecules caveolin-1 [17] and CD4 [18], the BRCA2 and CDKN1A-interacting protein BCCP, the Target of Rapamycin complex 2 subunit MAPKAP1 [18], and the lysosomal-associated protein LAPTM4B-35 [19]. Among these interactors, caveolin-1 [17] and RNF2 [11] negatively modulate Pgp activity, whereas LAPTM-35 is the only protein that has been shown to increase Pgp activity [19].

Here, we applied the Cell Surface Capturing (CSC) technology to investigate the surfaceome of a Pgp-negative (chemosensitive) and Pgp-positive (chemoresistant) human colon cancer model system, with the goal to identify quantitative surfaceome changes indicative of chemoresistance. This analysis identified carbonic anhydrase type XII (CAXII; accession number O43570, UniProtKB; http://www.uniprot.org) as a protein with significantly higher expression on the surface of chemoresistant cells. Based on this finding, we investigated whether changes in CAXII cell surface abundance were able to induce and/or maintain the MDR phenotype and whether CAXII could potentially be exploited as therapeutic target to chemosensitize MDR cells.

## RESULTS

### CAXII shows a higher cell surface abundance in chemoresistant cells than in chemosensitive cells

**Figure 1:**
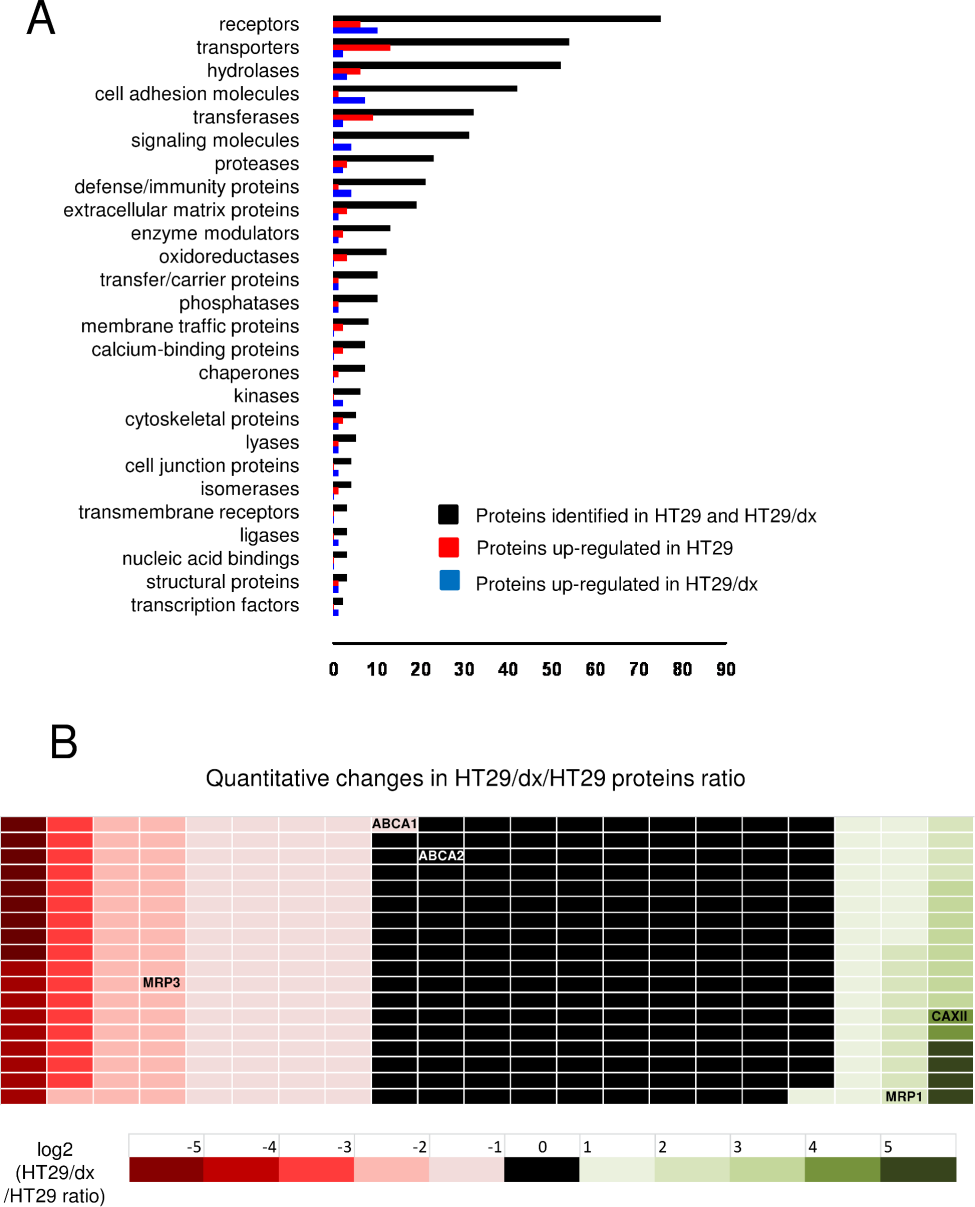
CSC technology enables the identification and quantitative comparison of the surface glycoproteome of human chemosensitive and chemoresistant colon cancer cells **(A)** 380 identified plasma membrane-associated proteins (black bars) from human chemosensitive colon cancer HT29 cells and human chemoresistant colon cancer HT29/dx cells were categorized according to the biological function assigned by the PANTHER algorithm. **(B)** Quantitative analysis of the proteins detected by CSC technology. The ratio between protein expressed in HT29/dx cells and protein expressed in HT29 cells is represented by a colorimetric logarithmic scale. CAXII, ABCC1/MRP1, ABCC3/MRP3, ABCA1 and ABCA2 hits are indicated.

CSC technology, which selectively tags and purifies cell surface exposed glycopeptides for analysis by mass spectrometry, enabled the identification of 380 cell surface residing glycoproteins from human chemosensitive colon cancer HT29 cells and human chemoresistant colon cancer HT29/dx cells. The functional classification of the identified proteins is shown in Figure 1A. The quantitative analysis of cell surface protein expression in HT29/dx and in HT29 cells is reported in Figure 1B and in Supplemental Table 1.

**Figure 2:**
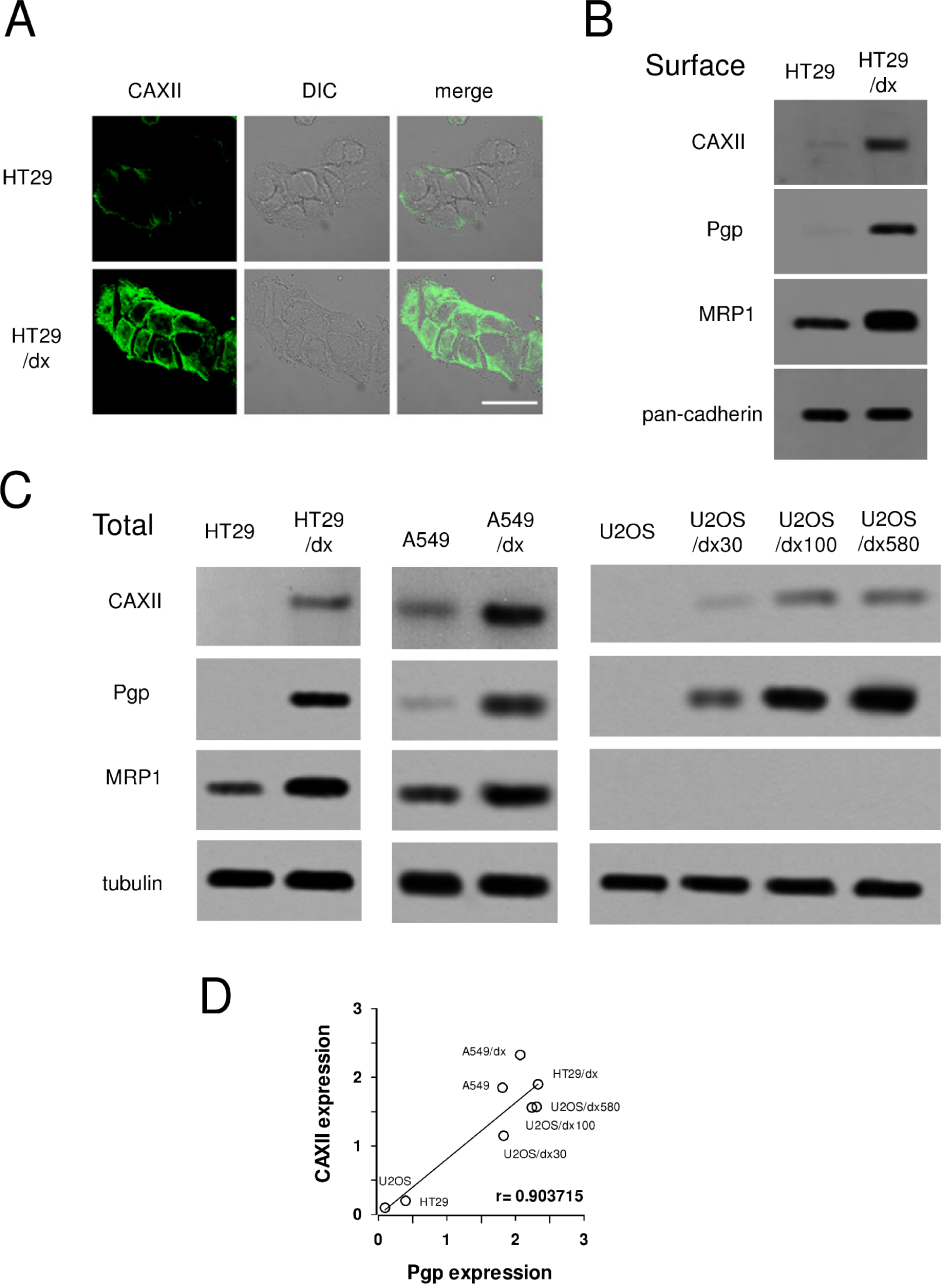
Expression of CAXII in chemosensitive and chemoresistant human cancer cells Human chemosensitive colon cancer HT29 cells and their chemoresistant counterpart HT29/dx cells, human chemosensitive lung cancer A549 cells and their chemoresistant counterpart A549/dx cells, human chemosensitive osteosarcoma U2-OS cells and the chemoresistant clones U2-OS/dx 30, U2-OS/dx 100, U2-OS/dx 580 were subjected to the following assays. **(A)** Confocal microscope analysis of HT29 and HT29/dx cells stained for CAXII. The samples were analyzed by laser scanning confocal microscope for green fluorescence signal (CAXII) or by Nomarski differential interference contrast (DIC) optics. Magnification: 60 × objective; 10 × ocular lens. Bar = 20 μm. **(B)** Western blot analysis of biotinylated plasma membrane associated CAXII, Pgp and MRP1 in HT29 and HT29/dx cells. The pan-cadherin expression was used as a control of equal protein loading. The figure is representative of three experiments with similar results. **(C)** Whole cell lysates were analyzed by Western blotting for the expression of CAXII, Pgp and MRP1. The β-tubulin expression was used as a control of equal protein loading. The figure is representative of three experiments with similar results. **(D)** Linear regression analysis between CAXII and Pgp expression. The mean band density of CAXII and Pgp (panel C), expressed as arbitrary units, was calculated by ImageJ software (http://www.rsb.info.nih.gov/ij/). r coefficient was calculated using Fig. P software (Fig. P Software Inc., Hamilton, Canada).

Among the ABC transporters detected on the surface of chemoresistant cells, ABCC1/MRP1 was more abundant on HT29/dx cells, and ABCC3/MRP3, ABCA1, ABCA2 showed lower expression levels. One of the proteins with highest relative expression in chemoresistant cells was CAXII, which was 16-fold more expressed in HT29/dx cells than in parental HT29 cells (Figure 1B and Supplemental Table 1). CAXII topology and identified glycopeptides are shown in Supplemental Figure 1. These data were confirmed by confocal microscopy analysis (Figure 2A) and by immunoblot analysis of biotinylated extracts from HT29 and HT29/dx cells (Figure 2B). Biotinylation assays confirmed that Pgp and MRP1 were more abundant on the surface of HT29/dx compared to HT29 cells (Figure 2B). The ratio of N-glycosylated to deglycosylated Pgp in plasma membrane extracts was about 1 in HT29/dx cells, whereas the ratio of N-glycosylated to deglycosylated MRP1 was higher than 1 (Supplemental Figure 2A and 2B).

The higher surface abundance level of CAXII was not due to differential trafficking, since intracellular pools of CAXII were also elevated in HT29/dx whole cell lysate (Figure 2C). This phenomenon was not specific to colon cancer cells and higher levels of CAXII were also found in chemoresistant lung cancer A549/dx cells compared to chemosensitive A549 cells (Figure 2C). Similarly, a comparison of U2-OS cells with corresponding doxorubicin-resistant clones (U2-OS/dx 30, U2-OS/dx 100, U2-OS/dx 580) revealed that CAXII levels also correlated with chemoresistance in these osteosarcoma cells (Figure 2C). All tested chemoresistant cell lines had higher expression of Pgp than the corresponding chemosensitive cells; HT29/dx and A549/dx cells also had higher levels of MRP1, which was undetectable in the chemoresistant osteosarcoma cells (Figure 2C). On the basis of these results, we found a strong direct correlation between CAXII and Pgp expression (Figure 2D); no correlation was found for CAXII and MRP1 expression (not shown). According to tissues expression data, CAXII and Pgp (Supplemental Figure 3 and 4), but not CAXII and MRP1 (Supplemental Figure 3 and 5), were expressed at similar levels in colon adenocarcinoma samples.

### CAXII expression increases during the acquisition of chemoresistance, in parallel with the increase of HIF-1α and Pgp

**Figure 3:**
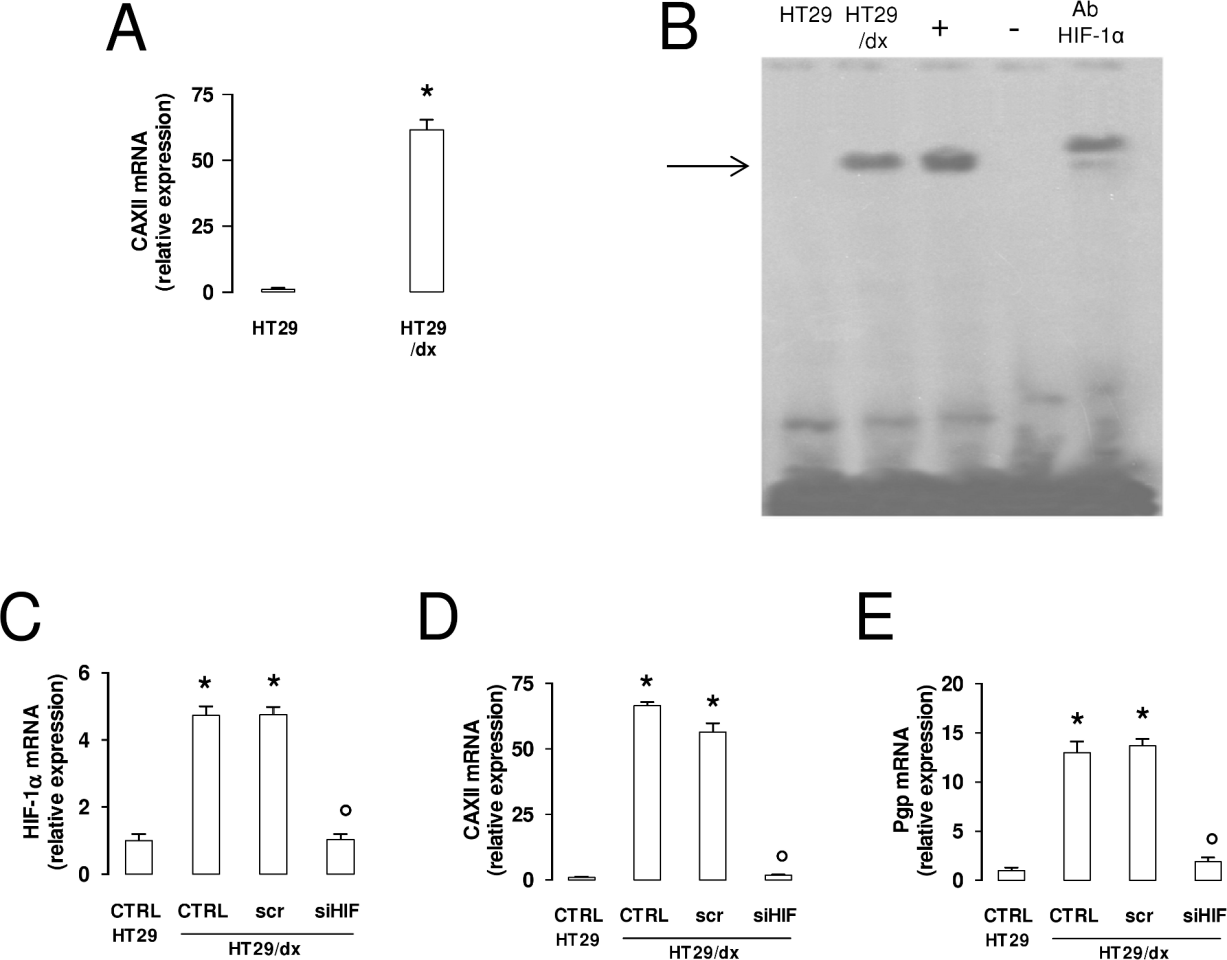
CAXII and Pgp expression levels are affected by HIF-1α in chemoresistant cells **(A)** The *CAXII* mRNA level in HT29 and HT29/dx cells was detected by qRT-PCR. Data are presented as means ± SD (*n* = 4). Versus HT29: *p < 0.001. **(B)** EMSA detection of HIF-1α bound to its DNA consensus sequence was performed on nuclear extracts of normoxic HT29 and HT29/dx cells. Hypoxic HT29 cells (grown at 2% O_2_ for 24 h) were used as positive control of HIF-1α activation (+). One lane was loaded with distilled water in place of cell extracts and was used as negative control (−). As control of specificity, the nuclear extracts of hypoxic HT29 cells were incubated with an anti-HIF-1α antibody (Ab HIF-1α). The band corresponding to the HIF-1α-DNA complex is indicated by the arrow. The figure is representative of three experiments with similar results. **(C–E)** mRNA was extracted from wild-type HT29 cells and HT29/dx cells (CTRL), HT29/dx cells treated with a non targeting scrambled siRNA (scr) or with a HIF-1α-targeting specific siRNA pool (siHIF) for 24 h. The expression of *HIF-1α* (panel **C**), *CAXII* (panel **D**) and *Pgp* (panel **E**) was detected by qRT-PCR. Data are presented as means ± SD (*n* = 4). Versus CTRL HT29: *p < 0.001; versus CTRL HT29/dx: ° *p* < 0.001.

The difference in CAXII protein expression between HT29 and HT29/dx cells was paralleled by a striking difference in mRNA levels (Figure 3A). Upstream regulatory regions of the *CAXII* gene contain hypoxia-response element (HRE) sequences [20], suggesting that the transcription factor hypoxia inducible factor-1α (HIF-1α) might be involved in the control of CAXII expression. HIF-1α activity was undetectable in HT29 cells, but present in HT29/dx where the protein was bound to HRE-containing DNA probes even under normoxic conditions (Figure 3B). In the chemoresistant cells, this leads to increased transcription of HIF-1α target genes, such as glucose transporter 1, hexokinase, aldolase-A, glyceraldehyde 3-phosphate dehydrogenase, phosphoglycerate kinase, enolase-A, lactate dehydrogenase, vascular endothelial growth factor, erythropoietin in the chemoresistant cells (Supplemental Figure 6). Moreover, HT29/dx cells had significantly higher levels of *HIF-1α* mRNA, together with increased levels of *CAXII* and *Pgp* mRNA, a known target gene of HIF-1α [21], than HT29 cells (Figure 3C–3E). Interestingly, *HIF-1α* silencing in HT29/dx cells (Figure 3C) produced a strong reduction of both *CAXII* (Figure 3D) and *Pgp* mRNA (Figure 3E), without affecting cell proliferation, apoptosis and viability of these cells (not shown).

**Figure 4:**
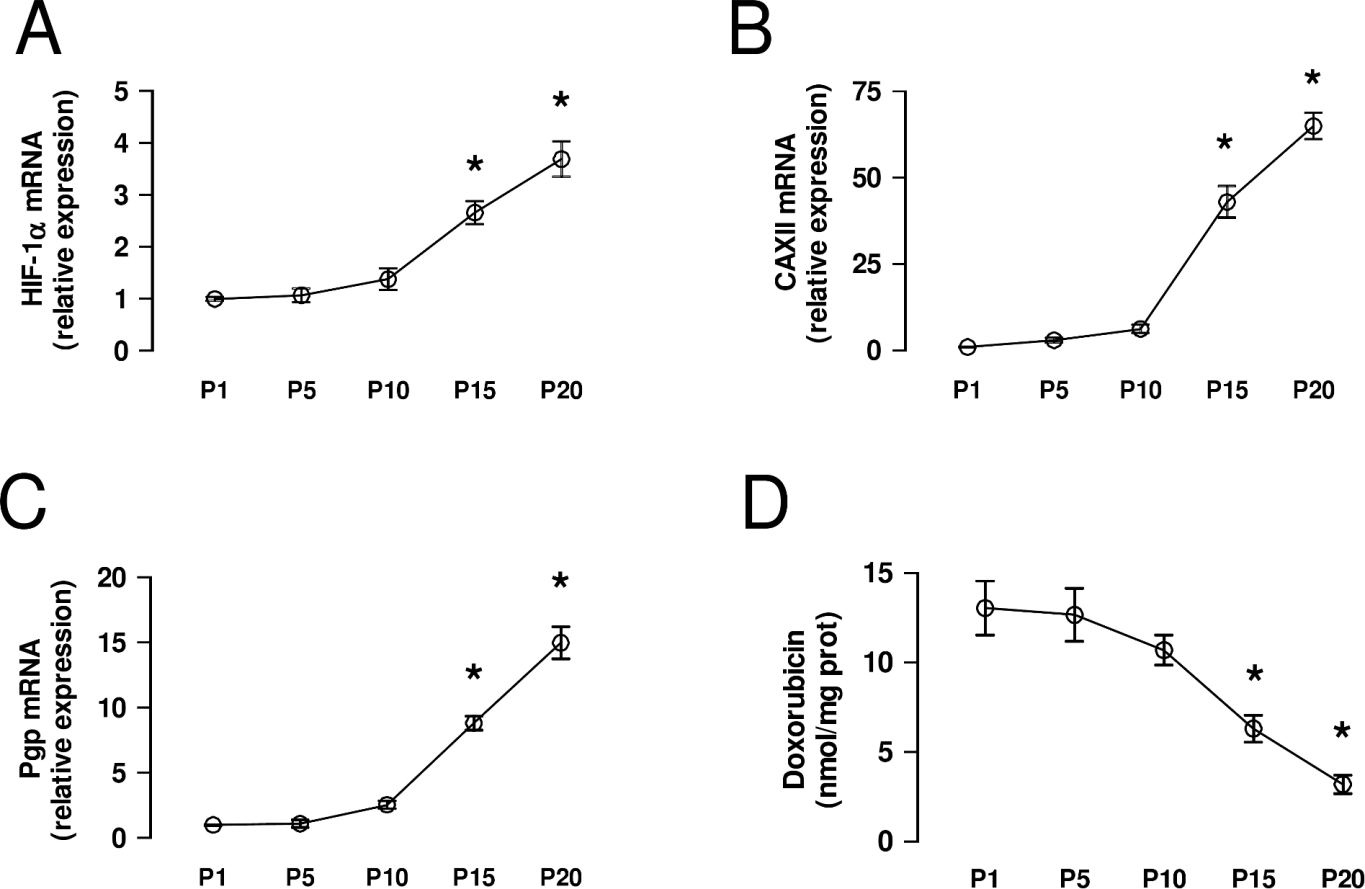
CAXII increases during the acquisition of chemoresistance HT29 cells were cultured in medium containing increasing concentrations of doxorubicin, as detailed under Methods. **(A–C)** At passage *(P)* 1, 5, 10, 15, 20 the mRNA was extracted and the expression of *HIF-1α* (panel **A**), *CAXII* (panel **B**) and *Pgp* (panel **C**) was detected by qRT-PCR. Data are presented as means ± SD (*n* = 4). Versus P1: *p < 0.001. **(D)** An aliquot of cells was incubated 24 h with 5 μmol/L doxorubicin, then lysed and analyzed for the intracellular doxorubicin content. Data are presented as means ± SD (*n* = 4). Versus P1: *p < 0.001.

The selection of chemoresistant cells from parental chemosensitive HT29 cells with increasing concentrations of doxorubicin induced a progressive increase of *HIF-1α* mRNA, measured every 5 passages of cell culture during the selection process (Figure 4A). The observed HIF-1α increase was paralleled by the progressive increase in *CAXII* (Figure 4B) and *Pgp* (Figure 4C) mRNA, and by the progressive decrease in the accumulation of doxorubicin (Figure 4D), a substrate of Pgp.

### Depletion of CAXII does not affect proliferation and survival of chemoresistant cells

**Figure 5:**
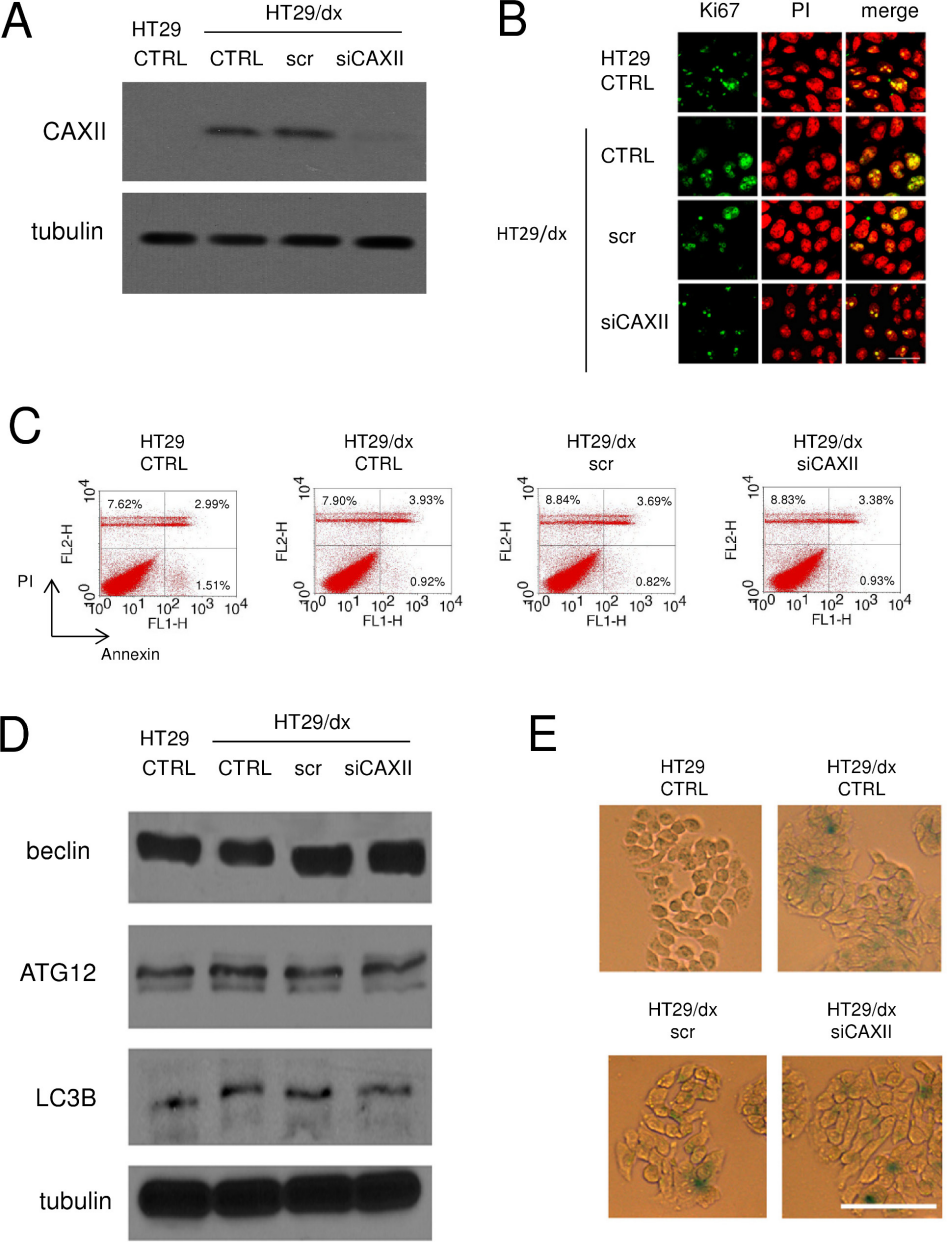
Depletion of CAXII does not affect proliferation and survival of chemoresistant cells HT29/dx cells were cultured for 48 h with fresh medium (CTRL), treated with a non targeting scrambled siRNA (scr) or with a CAXII-targeting specific siRNA pool (siCAXII). HT29 cells were included as control. **(A)** The expression of CAXII was measured in whole cell lysates by Western blotting. The β-tubulin expression was used as a control of equal protein loading. The figure is representative of three experiments with similar results. **(B)** Confocal microscope analysis of cells stained for the proliferation marker Ki67. The samples were analyzed by laser scanning confocal microscopy for Ki67 protein signal (green fluorescence) or for PI (red fluorescence), used to visualize nuclei. Magnification: 60 × objective; 10 × ocular lens. Bar = 20 μm. **(C)** The percentage of cells positive to annexin V-FITC, as index of early apoptosis, and to PI, as index of late apoptosis, was measured by flow cytometry. Percentages indicate annexin V-positive cells (lower right quadrant), PI-positive cells (upper left quadrant), annexin V/PI-positive cells (upper right quadrant). The figures are representative of three experiments with similar results. **(D)** Western blot analysis of the autophagy markers beclin, ATG12 and LC3B. The β-tubulin expression was used as a control of equal protein loading. The figure is representative of three experiments with similar results. **(E)** Cells were fixed and stained for β-galactosidase activity, then examined by fluorescence microscopy. Magnification: 20 × objective; 10 × ocular lens. Bar = 100 μm.

To investigate the functional role of CAXII in chemoresistant cells, we produced a HT29/dx subclone silenced for CAXII (Figure 5A). HT29 and HT29/dx cells did not show any appreciable difference in terms of: cell proliferation, as revealed by the proportion of Ki67-positive cells (Figure 5B); spontaneous apoptotic cell death, as indicated by the percentage of annexin V-fluorescein isothiocyanate (FITC)/propidium iodide (PI)-positive cells (Figure 5C); autophagy, as indicated by the expression level of classical autophagic markers such as beclin, ATG12 and LC3B (Figure 5D). Interestingly, untreated HT29/dx cells appeared more senescent than parental HT29 cells, as suggested by higher staining with β-galactosidase (Figure 5E). Despite the documented role of CAXII as a pro-oncogenic factor [22], enzyme silencing did not alter any of these parameters in chemoresistant cells (Figure 5B–5E).

### CAXII is associated with Pgp and is necessary to maintain Pgp-mediated chemoresistance

**Figure 6:**
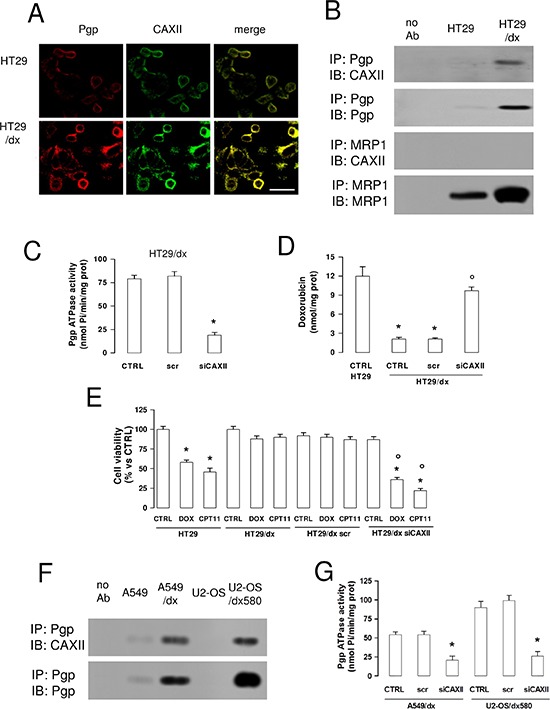
CAXII is physically associated with Pgp and increases Pgp activity in chemoresistant cells **(A)** Confocal microscope analysis of HT29 and HT29/dx cells stained for CAXII and Pgp. The samples were analyzed by laser scanning confocal microscope for green (CAXII) or red (Pgp) fluorescence signal. Magnification: 60 × objective; 10 × ocular lens. Bar = 20 μm. **(B)** Biotinylated plasma membrane-derived extracts from HT29 and HT29/dx cells were immunoprecipitated (IP) with anti-Pgp or anti-MRP1 antibodies, then immunoblotted (IB) with anti-CAXII, anti-Pgp or anti-MRP1 antibodies. no Ab: samples immunoprecipitated without antibody. The figure is representative of three experiments with similar results. **(C)** HT29/dx cells were cultured for 48 h with fresh medium (CTRL), treated with a non targeting scrambled siRNA (scr) or with a CAXII-targeting specific siRNA pool (siCAXII). The Pgp ATPase activity was measured spectrophotometrically on Pgp-rich vesicles extracted from membrane fractions. Data are presented as means ± SD (*n* = 4). Versus CTRL: *p < 0.001. **(D)** Cells treated as reported in C were incubated for 24 h with 5 μmol/L doxorubicin, then the intracellular drug content was measured fluorimetrically. HT29 were included as control of chemosensitive cells. Data are presented as means ± SD (*n* = 4). Versus HT29 CTRL: *p < 0.001; versus HT29/dx CTRL: ° *p* < 0.001. **(E)** Cells treated as reported in C were grown for 72 h in fresh medium (CTRL), or in medium containing 5 μmol/L doxorubicin (DOX) or 1 μM irinotecan (CPT11), then stained with neutral red dye. The absorbance of viable cells was measured spectrophotometrically. Data are presented as means ± SD (*n* = 4). For HT29 and HT29/dx cells, versus CTRL: *p < 0.001; for HT29/dx cells, versus wild type HT29/dx DOX or HT29/dx CPT11, respectively: ° *p* < 0.002. **(F)** Biotinylated plasma membrane-derived extracts from human chemosensitive lung cancer A549 cell and chemoresistant A549/dx cells, human chemosensitive osteosarcoma U2-OS cells and chemoresistant U2-OS/dx580 cells were immunoprecipitated (IP) with an anti-Pgp antibody, then immunoblotted (IB) with an anti-CAXII or an anti-Pgp antibody. no Ab: samples immunoprecipitated without antibody. The figure is representative of two experiments with similar results. **(G)** A549/dx and U2-OS/dx580 cells were grown in fresh medium (CTRL), treated with a non targeting scrambled siRNA (scr) or with a CAXII-targeting specific siRNA pool (siCAXII). The Pgp ATPase activity was measured spectrophotometrically on Pgp-rich vesicles extracted from membrane fractions. Data are presented as means ± SD (*n* = 3). For both cell lines, versus CTRL: *p < 0.001.

Confocal microscope analysis showed that CAXII and Pgp co-localized on HT29/dx cells plasma membrane (Figure 6A). In co-immunoprecipitation assays, we found that CAXII was physically associated with Pgp, but not with MRP1 on HT29/dx cells plasma membrane (Figure 6B). CAXII co-immunoprecipitated with both glycosylated and deglycosylated Pgp (Supplemental Figure 7A). In some cell lines deglycosylated Pgp is less active, but in HT29/dx the glycosylation status of Pgp did not affect its ATPase activity (Supplemental Figure 7B).

These observations raised the question whether CAXII directly affected the activity of Pgp. Supporting this hypothesis, CAXII silencing in HT29/dx cells led to a dramatic decrease in Pgp ATPase activity (Figure 6C). As expected, HT29/dx cells accumulated significantly less doxorubicin than HT29 cells. In contrast, CAXII-silenced HT29/dx cells showed significantly increased levels of intracellular doxorubicin, reaching the same amount measured in HT29 cells (Figure 6D), where Pgp was undetectable (Figure 2C). In keeping with the different expression levels of Pgp, chemotherapeutic drugs that are substrates of Pgp, such as doxorubicin and irinotecan [23], reduced cell viability of HT29 cells, but not HT29/dx cells. However, both doxorubicin and irinotecan exhibited cytotoxic effects on HT29/dx cells silenced for CAXII (Figure 6E).

The association between Pgp and CAXII was shared by other chemoresistant cells, such as lung cancer A549/dx cells and osteosarcoma resistant clones (Figure 6F). In analogy to HT29/dx cells, CAXII silencing (Supplemental Figure 8) reduced Pgp ATPase activity in these cell lines (Figure 6G).

**Figure 7:**
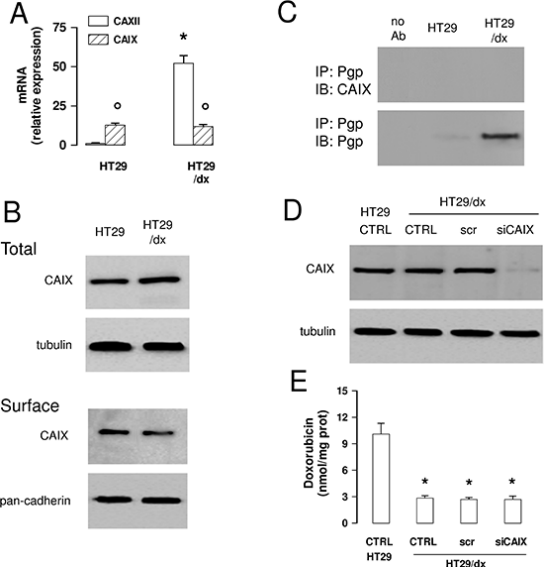
CAIX does not mediate chemoresistance to Pgp substrates in colon cancer cells **(A)** The *CAIX* mRNA level in HT29 and HT29/dx cells was detected by qRT-PCR. *CAXII* mRNA level is shown for comparison. Data are presented as means ± SD (*n* = 4). Versus HT29: *p < 0.001; for both HT29 and HT29/dx cells, *CAIX* versus *CAXII* expression: ° *p* < 0.001. **(B)** Western blot analysis of CAIX expression in whole cell lysates (upper panel; Total) and biotinylated plasma membrane-derived extracts (lower panel; Surface) in HT29 and HT29/dx cells. The β-tubulin and pan-cadherin expression were used as controls of equal protein loading in whole cell lysates and plasma membrane-derived extracts, respectively. The figure is representative of three experiments with similar results. **(C)** Biotinylated plasma membrane-derived extracts from HT29 and HT29/dx cells were immunoprecipitated (IP) with an anti-Pgp antibody, then immunoblotted (IB) with an anti-CAIX or an anti-Pgp antibody. no Ab: samples immunoprecipitated without antibody. The figure is representative of three experiments with similar results. **(D)** HT29/dx cells were cultured for 48 h with fresh medium (CTRL), treated with a non targeting scrambled siRNA (scr) or with a CAIX-targeting specific siRNA pool (siCAIX). HT29 cells were included as control. The expression of CAIX was measured in whole cell lysates by Western blotting. The β-tubulin expression was used as a control of equal protein loading. The figure is representative of three experiments with similar results. **(E)** HT29/dx cells treated as reported in D were incubated for 24 h with 5 μmol/L doxorubicin, then the intracellular drug content was measured fluorimetrically. HT29 cells were included as control of chemosensitive cells. Data are presented as means ± SD (*n* = 4). Versus HT29 CTRL: *p < 0.001.

CAIX is another isoform of carbonic anhydrase. This isoform is known to be highly expressed in tumors, including colon cancer, and has been linked to tumor aggressiveness and resistance to therapy [20]. This led us to investigate the expression levels of CAIX in our model system. In HT29 cells, *CAIX* mRNA levels were higher than the ones of *CAXII*, but were not increased in HT29/dx cells, where levels were significantly lower than for *CAXII* (Figure 7A). CAIX protein was detectable in HT29 and HT29/dx cells, without differences between chemosensitive and chemoresistant cells, both in whole cell lysates and in plasma membrane-derived extracts (Figure 7B). In contrast to CAXII (Figure 6B), CAIX did not co-immunoprecipitate with Pgp in plasma membrane extracts (Figure 7C). Moreover, CAIX-silenced HT29/dx cells (Figure 7D) retained the same low amount of intracellular doxorubicin than wild-type HT29/dx cells (Figure 7E), suggesting that CAIX is not involved in the chemosensitization towards Pgp substrates.

### Pharmacological inhibition of CAXII restores chemosensitivity in chemoresistant cancer cells

**Figure 8:**
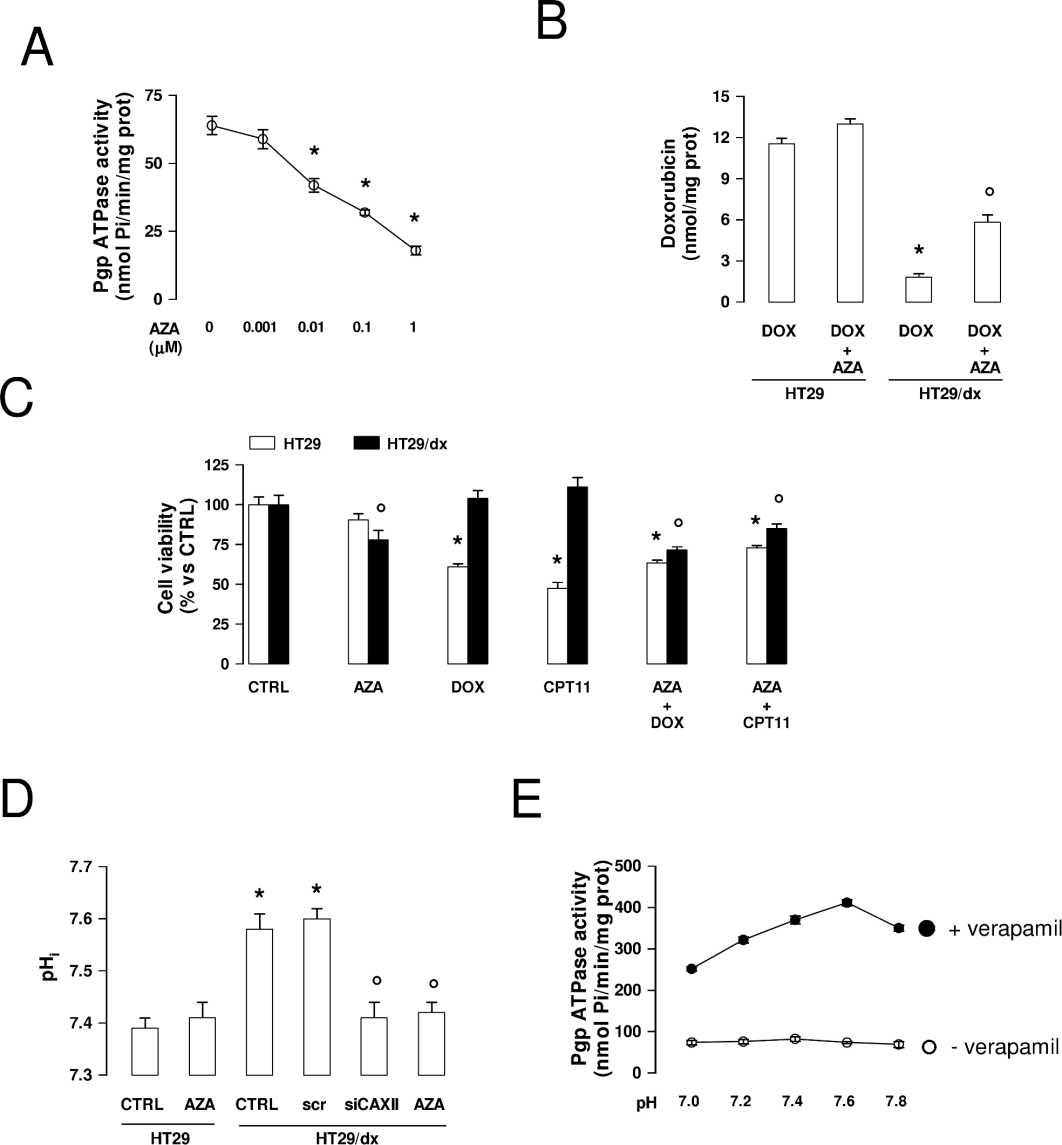
Effects of acetazolamide on Pgp activity, chemosensitivity and intracellular pH in chemoresistant cancer cells **(A)** Pgp-rich vesicles were extracted from membrane fractions of HT29/dx cells, then the Pgp ATPase activity was measured spectrophotometrically in the absence (0) or in the presence of increasing concentrations of acetazolamide (AZA), added during the assay. Data are presented as means ± SD (*n* = 4). Versus 0: *p < 0.005. **(B)** HT29 and HT29/dx were incubated for 24 h with 5 μmol/L doxorubicin (DOX). When indicated, 1 μmol/L acetazolamide (AZA) was co-incubated. The intracellular doxorubicin retention was measured fluorimetrically. Data are presented as means ± SD (*n* = 4). HT29/dx DOX cells versus HT29 DOX cells: *p < 0.001; HT29/dx DOX+AZA cells versus HT29/dx DOX cells: ° *p* < 0.005. **(C)** Cells were grown for 72 h in fresh medium (CTRL), or in medium containing 1 μM acetazolamide (AZA), 5 μmol/L doxorubicin (DOX), 1 μM irinotecan (CPT11), alone or in combination, then stained with neutral red dye. The absorbance of viable cells was measured spectrophotometrically. Data are presented as means ± SD (*n* = 4). For HT29 cells, versus CTRL: *p < 0.001; for HT29/dx cells, versus CTRL: ° *p* < 0.05. **(D)** The intracellular pH (pH_i_) measurement was performed in duplicate (*n* = 4) on HT29 and HT29/dx cells untreated or treated with a non targeting scrambled siRNA (scr), with a CAXII-targeting specific siRNA pool (siCAXII) for 48 h, with 1 μmol/L acetazolamide (AZA) for 24 h. Significance versus HT29 cells: *p < 0.01; versus HT29/dx cells: ° *p* < 0.02. **(E)** The Pgp ATPase activity was measured spectrophotometrically in Pgp-rich vesicles extracted from HT29/dx cell membrane fractions, using buffers with different pH, in the absence (open circles) or presence (solid circles) of 10 μmol/L verapamil, chosen as a Pgp activator. Data are presented as means ± SD (*n* = 4).

Similarly to the effect of CAXII silencing, the CAXII inhibitor acetazolamide (Ki 5.6 ± 0.2 nM) [24] dose-dependently reduced the activity of Pgp (Figure 8A), suggesting that the activity of CAXII is critical for the catalytic activity of the transporter. At 1 μmol/L, acetazolamide, which was devoid of effects in HT29 cells where CAXII and Pgp were hardly detectable by Western blot (Figure 2B–2C), significantly increased the retention of doxorubicin (Figure 8B) and restored the cytotoxic effects of doxorubicin and irinotecan in HT29/dx cells (Figure 8C).

The activity of CAIX and CAXII has been reported to induce intracellular alkalinization [25]. As shown in Figure 8D, the pH_i_ of HT29 was 7.39 ± 0.02, whereas the pHi of HT29/dx was 7.58 ± 0.03. To investigate whether CAXII contributes to this different pHi between chemosensitive and chemoresistant cells, we measured the pHi in HT29/dx cells silenced for CAXII or treated with acetazolamide: interestingly, these experimental conditions lowered the pH_i_ of HT29/dx cells to values similar to HT29 cells. By contrast, acetazolamide did not affect the pH_i_ of low CAXII-expressing HT29 cells (Figure 8D). To evaluate whether such different pHi conditions may affect the activity of Pgp, we measured the ATPase activity of Pgp from HT29/dx plasma membrane in buffers with different pH. We did not detect changes in the basal ATPase activity of Pgp; by contrast, the verapamil-stimulated ATPase activity of Pgp, taken as an index of the transporter's maximal activity, increased from pH 7.0 to pH 7.6 (Figure 8E), suggesting that the optimal pH at which Pgp operates is slightly alkaline (i.e. compatible with the pH_i_ of HT29/dx cells) and that the pH produced by CAXII inhibition likely lowered Pgp activity.

## DISCUSSION

In this work we analyzed the surfaceome of chemosensitive Pgp-negative and chemoresistant Pgp-positive human colon cancer cells and identified CAXII more highly expressed in the latter.

CAXII is a surface-associated enzyme highly expressed in tumors of renal [26], ovarian [27] and colorectal [28] origin, where it maintains the homeostasis of HCO_3_^−^ [29].

In our model of chemoresistant colon cancer cells, CAXII was increased at both the protein and mRNA level, suggesting that *CAXII* gene transcription was up-regulated. The presence of HRE sequences in upstream regions of the *CAXII* gene, and the down-regulation of *CAXII* mRNA in cells expressing the Von Hippel Lindau protein, a HIF-1α-inhibitor [20], led us to the hypothesis that HIF-1α might be involved in the control of CAXII expression. In HT29/dx cells, HIF-1α was constitutively active even under normoxia, as demonstrated by its constitutive binding of hypoxia-responsive DNA elements, and by higher expression levels of its classical target genes. This characteristic was progressively acquired during the selection of resistant clones from the parental chemosensitive population exposed to doxorubicin. Although we have previously reported the constitutive activation of HIF-1α in normoxic cancer cells with a stable MDR phenotype [30], the parallel increase in HIF-1α transcription and the progressive acquisition of MDR is a new observation. This trend may be due to the use of doxorubicin as selective agent: indeed, in normoxia the drug produces reactive oxygen species [31] that increase HIF-1α [32]. During the onset of the MDR phenotype, the increase of HIF-1α was paralleled by an increase of *CAXII* and *Pgp* mRNAs. These data suggest that HIF-1α may play a role in the up-regulation of CAXII in HT29/dx cells. Since the relative increase of *CAXII* mRNA was greater than for classical target genes of HIF-1α, other transcription factors and co-activators induced during the selection of chemoresistant clones – in addition to HIF-1α – are likely to be involved in the up-regulation of the *CAXII* gene.

In previous reports, CAXII overexpression has been associated with good [33, 34] or poor [35, 36] prognosis, depending on the tumor type. Furthermore, the protein has been reported to support cell proliferation and invasion, based on the observations that a monoclonal antibody against CAXII reduced tumor growth [22] and that CAXII inhibitors lowered metastatic potential [37] in mouse xenografts. However, according to the results obtained by CAXII silencing in HT29/dx cells, CAXII did not confer any selective advantage in terms of cell proliferation, spontaneous necrotic/apoptotic death, autophagy or senescence rate. By contrast, we found that CAXII promoted the acquisition and maintenance of chemoresistance.

In our study, Pgp and MRP1 were found to be up-regulated on the plasma membrane of HT29/dx cells. Whereas CSC technology successfully identified MRP1, it failed to identify Pgp. The gel shift of the MRP1 band upon treatment with Peptide-N-Glycosidase F (PNGase F) was bigger than the gel shift of the Pgp band, suggesting that the extent of glycosylation was higher in MRP1. This makes MRP1 easier to detect by CSC, which specifically identifies N-glycopeptides of cell surface proteins. Moreover, Pgp was highly abundant in its deglycosylated form in HT29/dx cells. Of note, CAXII was physically associated with both glycosylated and deglycosylated Pgp, which were equally active. We did not further investigate which domain of CAXII may be responsible for the interaction with Pgp. Pgp has a large intracellular domain (Supplemental Figure 9) and according to the crystal structure of CAXII, the intracellular C-terminal domain of the enzyme is likely to be responsible for the interactions with other proteins, the oligomerization of CAXII, the catalytic activity and the signal transduction [38].

In all chemoresistant cells tested in this study, CAXII appeared to support Pgp activity: CAXII-silenced cells showed a decreased ATPase activity of Pgp, an increased retention of the Pgp substrate doxorubicin, and a restored cytotoxicity of doxorubicin and irinotecan.

However, we cannot exclude that other tumor-associated CA isoforms are involved in the onset or maintenance of chemoresistance. For instance, the overexpression of CAIX has been correlated with a low response to doxorubicin treatment in breast cancer patients [39]. In our model, CAIX was expressed at similar levels in HT29 and HT29/dx cells. Furthermore, it seemed to play a minor role in the resistance to doxorubicin as illustrated by the fact that CAIX-silenced HT29/dx cells had the same low intracellular retention of doxorubicin than wild-type HT29/dx cells. What about the resistance phenotype?

It has previously been reported that the verapamil-stimulated activity of Pgp, considered an index of the maximal activity of the transporter, increases at slightly alkaline pH [40]. We confirmed this trend in Pgp-rich vesicles extracted from HT29/dx cells. The pH_i_ measured in HT29/dx cells was lowered by CAXII silencing or by pharmacological CAXII inhibition with acetazolamide, suggesting that CAXII may contribute to maintain the slightly alkaline pH_i_ of chemoresistant cells. A slightly alkaline pHi has been shown to induce resistance to doxorubicin in HT29/dx cells [41]. Furthermore, it has been reported that the activity of both CAIX and CAXII regulate the pHi homeostasis in cancer cells [25]. In our experiments, the selective silencing of CAIX and CAXII in HT29/dx cells suggested that only the latter was involved in the maintenance of doxorubicin resistance in this model. Since CAIX did not physically interact with Pgp, we hypothesize that it did not produce a sufficient alkalinization to reduce the transporter's efflux activity in the plasma membrane area where Pgp is localized. By contrast, the co-localization of Pgp and CAXII may favor the optimal pH condition for Pgp activity in the microenvironment where Pgp and CAXII are concentrated.

Our work shows that pharmacological inhibition of CAXII with acetazolamide effectively sensitizes resistant cells to the cytotoxic effects of Pgp substrates. Acetazolamide has antitumor activity *in vivo* [42] and increases doxorubicin cytotoxicity in HT29 cells under hypoxia [43].

Since acetazolamide is being used in clinical practice as a diuretic, our work – although based on *in vitro* experiments – may have translational potential in the future. Given the role of CAXII in tumor growth and invasion, selective inhibitors of CAXII are under active development as new anticancer drugs [44–46], in particular against hypoxic tumors [47]. We propose that these synthetic and more selective inhibitors might be used as chemosensitizing agents for the treatment of Pgp overexpressing tumors. To achieve a more selective targeting of tumor cells, liposome- or nanoparticle-based carriers, which increase the intratumor accumulation of drugs, either by passive targeting (i.e. by the enhanced permeability retention effect) or active targeting [48], represent a valid approach. Our proteomic screening showed that, besides CAXII, a considerable number of other surface antigens were expressed at significantly higher levels in MDR cells. Some of these proteins may represent suitable targets for liposomes carrying CAXII inhibitors for the active and more selective targeting of chemoresistant colon cancer cells in preclinical models.

In summary, we show for the first time that CAXII is more highly abundant at the plasma membrane of Pgp-positive chemoresistant cells and is increased in parallel with Pgp during the acquisition of chemoresistance. Since CAXII activity is necessary for the optimal activity of Pgp, CAXII inhibitors may represent promising therapeutic tools to overcome Pgp-mediated chemoresistance.

## METHODS

### Chemicals

The plasticware for cell cultures was obtained from Falcon (Becton Dickinson, Franklin Lakes, NJ). The electrophoresis reagents were obtained from Bio-Rad Laboratories (Hercules, CA). The protein content of cell lysates was assessed with the BCA kit from Sigma Chemicals Co. (St. Louis, MO). Unless specified otherwise, all reagents were purchased from Sigma Chemicals Co.

### Cells

Human chemosensitive colon cancer HT29 cells (from ATCC, Manassas, VA) were cultured in RPMI 1640 medium. A subpopulation, called HT29/dx, was created by culturing parental cells with 12.5 nmol/L doxorubicin for passages 1–5, 25 nmol/L doxorubicin for passages 6–10, 50 nmol/L doxorubicin for passages 11–15, 100 nmol/L doxorubicin for passages 16–20, then stably maintaining cells in RPMI 1640 medium containing 200 nmol/L doxorubicin. HT29-dx cells displayed a higher abundance of Pgp, MRP1 and BCRP, and were cross-resistant to doxorubicin and irinotecan [49]. Human chemosensitive lung cancer A549 cells (ATCC) and the chemoresistant A549/dx cell subline were obtained and cultured as reported [50]. Human doxorubicin sensitive osteosarcoma U2-OS cells and the corresponding clones with increasing resistance to doxorubicin (U2-OS/dx 30, U2-OS/dx 100, U2-OS/dx 580), selected by culturing U2-OS cells in a medium with 30, 100, 580 ng/mL doxorubicin, were a kind gift from Dr. Massimo Serra, Laboratory of Experimental Oncology (IRCCS Istituto Ortopedico Rizzoli, Bologna, Italy), and have been previously characterized [51].

### Surface glycoprotein identification via CSC technology

Cells were prepared for CSC as described elsewhere [52]. Briefly, cells were treated for 15 min at 4°C in the dark with 2 mmol/L sodium meta-periodate (Thermo Fisher Scientific Inc., Waltham, MA) in PBS, pH 6.5, then washed and incubated with 6.5 mmol/L biocytin hydrazide (Biotium, Hayward, CA) in PBS, pH 6.5, for 1 h to biotinylate oxidized carbohydrates of cell surface glycoproteins. Cells were washed, incubated on ice in hypotonic lysis buffer (10 mmol/L Tris, 0.5 mmol/L MgCl_2_, 10 mmol/L iodoacetamide, pH 7.5) for 10 min, homogenized for 10 s using a Dounce homogenizer. Afterward cell debris and nuclei were removed by centrifugation at 1 700 × g for 7 min. The supernatant was solubilized in 400 μL of digestion buffer (100 mmol/L NH_4_HCO_3_, 1 mmol/L iodoacetamide, 1 mmol/L 2,2′-thiodiethanol, 0.1% w/v RapiGest; Waters, Milan, Italy) and sonicated with a VialTweeter instrument (Hielscher Ultrasonic GmbH, Teltow, Germany). Proteins were digested overnight with trypsin in a protease:protein ratio of 1:100. After protein digestion, the peptide mixture was heated at 95°C for 10 min to inactivate trypsin, then biotinylated glycopeptides were bound to Streptavidin Plus UltraLink Resin (SA beads; Pierce, Rockford, IL) for 3 h at 37°C. After extensive washing, cysteine containing peptides, which were bound via a disulfide bridge to the biotinylated glycopeptides, were eluted from the SA beads by incubation with elution buffer (100 mmol/L NH_4_HCO_3_, 10 mmol/L tris(2-carboxyethyl)phosphine, 1 mmol/L dithiothreitol) for 1 h at room temperature. SA beads were washed again and N-linked glycopeptides were enzymatically released from the SA beads in a second overnight elution step in the presence of PNGase F. The free thiols of cysteine containing peptides were alkylated with iodoacetamide. Peptides were desalted on Ultra MicroTIP Columns (The Nest Group, Southborough, MA) and dried in a SpeedVac concentrator. Finally, peptides were solubilized in LC-MS grade water containing 0.1% v/v formic acid and 5% v/v acetonitrile. MS/MS spectra were acquired with a LTQ Orbitrap XL mass spectrometer, converted to mzXML and searched against the UniProt database (Version 57.15) using the SEQUEST algorithm. Statistical data analysis was performed using a combination of ISB (Institute of Systems Biology, Seattle, WA) open-source software tools (PeptideProphetTM, ProteinProphetTM, TPP Version 4.3.1). A ProteinProphet probability score of at least 0.9 was used for data-filtering (corresponding to a false discovery rate of 1%). The protein functional classification was performed using the PANTHER algorithm (http://www.pantherdb.org). Quantitative data analysis was performed using the XPRESS software.

### Western blot analysis

For whole cell lysates, the cells were rinsed with ice-cold lysis buffer (50 mmol/L Tris, 10 mmol/L EDTA, 1% v/v Triton-X100), supplemented with the protease inhibitor cocktail set III (80 μmol/L aprotinin, 5 mmol/L bestatin, 1.5 mmol/L leupeptin, 1 mmol/L pepstatin; Calbiochem, San Diego, CA), 2 mmol/L phenylmethylsulfonyl fluoride and 1 mmol/L Na_3_VO_4_, then sonicated and centrifuged at 13 000 × g for 10 min at 4°C. 20 μg protein extracts were subjected to SDS-PAGE and probed with the following antibodies: anti-CAXII (Abcam, Cambridge, UK); anti-CAIX (Novus Biologicals, Littleton, CO); anti-Pgp (C219, Calbiochem); anti-MRP1 (Abcam); anti-beclin (Abcam); anti-ATG12 (Abcam); anti-LC3B (Abcam); anti-β-tubulin (Santa Cruz Biotechnology Inc., Santa Cruz, CA), followed by a peroxidase-conjugated secondary antibody (Bio-Rad Laboratories). The membranes were washed with Tris-buffered saline-Tween 0.1% v/v, and the proteins were detected by enhanced chemiluminescence (Bio-Rad Laboratories). Plasma membrane-associated CAXII was evaluated in biotinylation assays, using the Cell Surface Protein isolation kit (Thermo Fisher Scientific Inc.), as previously reported [53], using an anti pan-cadherin antibody (Santa Cruz Biotechnology Inc.) to check the equal protein loading. In co-immunoprecipitation experiments, 100 μg of proteins were immunoprecipitated with the anti-Pgp or anti-MRP1 antibodies, using the PureProteome protein A and protein G Magnetic Beads (Millipore, Billerica, MA). The immunoprecipitated proteins were separated by SDS-PAGE and probed with the anti-CAXII, anti-CAIX, anti-Pgp or anti-MRP1 antibodies, followed by a peroxidase-conjugated secondary antibody. To detect glycosylated and deglycosyated proteins, biotinylated extracts (100 μg) were heated at 99°C for 10 min to denature proteins, then incubated for 1 h at 37°C in the absence or presence of 1 μU of human recombinant PNGaseF. Samples were resolved by SDS-PAGE and probed with the following antibodies, recognizing both the glycosylated and the deglycosylated forms: anti-Pgp (3C3.2, Millipore); anti-MRP1 (Enzo Life Sciences, Farmingdale, NY).

### Confocal microscopy analysis

5 × 10^5^ cells were grown on sterile glass coverslips, rinsed and fixed with 4% w/v paraformaldehyde for 15 min. To visualize surface CAXII and Pgp, the samples were washed with PBS and stained with an anti-CAXII antibody or an anti-Pgp antibody conjugated to phycoerythrin (Millipore) for 1 h. After washing, samples stained for CAXII were incubated with an AlexaFluor 488-conjugated secondary antibody (Millipore) for 1 h and re-washed. The coverslips were mounted with 4 μL of Gel Mount Aqueous Mounting and examined with an Olympus FV300 laser scanning confocal microscope (Olympus Biosystems, Tokyo, Japan). For each experimental point, a minimum of five microscopic fields were examined.

### Quantitative real time-PCR (qRT-PCR)

Total RNA was extracted and reverse-transcribed using the QuantiTect Reverse Transcription Kit (Qiagen, Hilden, Germany). The qRT-PCR was performed with the IQ™ SYBR Green Supermix (Bio-Rad Laboratories). The same cDNA preparation was used to quantify the genes of interest and the housekeeping gene *S14*. The primer sequences, designed using the qPrimerDepot database (http://primerdepot.nci.nih.gov/), are reported in the Supplemental Table 2. The relative quantification was performed by comparing each PCR product with the housekeeping PCR product, using the Bio-Rad Software Gene Expression Quantitation (Bio-Rad Laboratories).

### Electrophoretic mobility shift assay (EMSA)

Nuclear extracts were prepared as previously reported [54]. The probe containing the HIF-1α oligonucleotide consensus sequence was labeled with [γ-^32^P]-ATP (3,000 Ci/μmol, 250 mCi; Amersham International, Little Chalfont, UK), using T4 polynucleotide kinase (Roche, Basel, Switzerland). Oligonucleotide sequence was 5′-TCTGTACGTGACCACACTCACCTC-3′. For each extract, 10 μg was incubated for 20 min with 20 000 cpm of [^32^P]-labeled double-stranded oligonucleotide at 4°C. In the supershift assay, nuclear extracts were pre-incubated for 30 min at room temperature with 2 μL of an anti-HIF-1α antibody (Millipore); the reaction mixture containing the [^32^P]-labeled double-stranded oligonucleotide was then added. The DNA-protein complex was separated on a non denaturating 4% polyacrylamide gel in TBE buffer (0.4 mol/L Tris, 0.45 mol/L boric acid, 0.5 mol/L EDTA, pH 8.0). After electrophoresis, the gel was dried and autoradiographed by exposure to X-ray film for 24 h.

### Gene silencing

2 × 10^5^ cells were transfected with 400 nmol/L of 20–25 nucleotide non targeting scrambled siRNA (Control siRNA-A, Santa Cruz Biotechnology Inc.) or specific siRNA pools for CAXII, CAIX or HIF-1α (Santa Cruz Biotechnology Inc.), following the manufacturer's instructions. To verify the silencing efficacy, 24 h after the transfection the levels of mRNAs were checked by qRT-PCR, 48 h after the transfection the expression of proteins was checked by Western blotting. To verify the absence of cell toxicity after silencing, cell proliferation, apoptosis and viability was measured, as reported below.

### Cell proliferation, apoptosis and senescence

To evaluate cell proliferation, 5 × 10^5^ cells were grown on sterile glass coverslips, rinsed and fixed with 4% w/v paraformaldehyde for 15 min, then permeabilized with 0.1% v/v Triton-X100 for 5 min on ice, washed three times with PBS and stained with an anti-Ki67 antibody (Abcam) for 1 h at room temperature. After washing, samples were incubated with an AlexaFluor 488-conjugated secondary antibody (Millipore) for 1 h and re-washed. Finally, cells were stained with PI (1 μg/ml) to counterstain the nuclei, and washed again. The coverslips were mounted with 4 μL of Gel Mount Aqueous Mounting and examined by confocal microscopy as detailed above. Early and late apoptosis was measured by the Annexin V/Propidium Iodide Apoptosis Detection Kit (Sigma Chemical Co.). 1 × 10^5^ cells were analyzed with a FACS-Calibur flow cytometer (Becton Dickinson). The percentage of cells positive to annexin V-FITC and PI was calculated with the Cell Quest software (Becton Dickinson). Cell senescence was evaluated on 5 × 10^5^ cells fixed and stained with the Senescence Cells Histochemical Staining Kit (Sigma Chemical Co.), following the manufacturer's instruction. Samples were examined with a Leica DC100 fluorescence microscope (Leica Microsystems GmbH, Wetzlar, Germany). For each experimental point, a minimum of five microscopic fields were examined.

### Intracellular doxorubicin accumulation

Doxorubicin content was measured fluorimetrically as detailed elsewhere [55]. The results were expressed as nmol doxorubicin/mg cell proteins, according to a titration curve previously set.

### Cell viability

Cell viability was evaluated by measuring the percentage of cells stained with neutral red dye, as reported previously [49]. The viability of untreated cells was considered 100%; the results were expressed as percentage of viable cells in each experimental condition versus untreated cells.

### ATPase Pgp activity

The assay was performed on Pgp-enriched membrane vesicles as detailed elsewhere [56]. Verapamil (10 μmol/L) was added to the reaction mix to achieve a maximal activation of the Pgp ATPase activity. Results were expressed as nmol hydrolyzed phosphate (Pi)/min/mg proteins, according to the titration curve previously prepared.

### Intracellular pH (pH_i_) measurement

The pHi was measured by incubating whole cells with 5 μmol/L of 2′,7′-bis-(2-carboxyethyl)-5-(and-6)-carboxyfluorescein acetoxymethyl ester (BCECF-AM) for 15 min at 37°C and reading the intracellular fluorescence by a FACSCalibur flow cytometer (Becton Dickinson). The intracellular fluorescence was converted into pH units according to a titration curve, as described previously [41].

### Statistical analysis

All data in text and figures are provided as means ± SD. The results were analyzed by a one-way Analysis of Variance (ANOVA). A *p* < 0.05 was considered significant.

## SUPPLEMENTARY FIGURES AND TABLES




