# Supplementary material for: Carbonic anhydrase XII is a new therapeutic target to overcome chemoresistance in cancer cells
Source: Oncotarget. 2015 Feb 18;6(9):6776–93. doi: 10.18632/oncotarget.2882 (PMC4466649; doi:10.18632/oncotarget.2882)
Supplement: Supplementary file 1 [file oncotarget-06-6776-s001.pdf]

SUPPLEMENTARY FIGURES AND TABLES

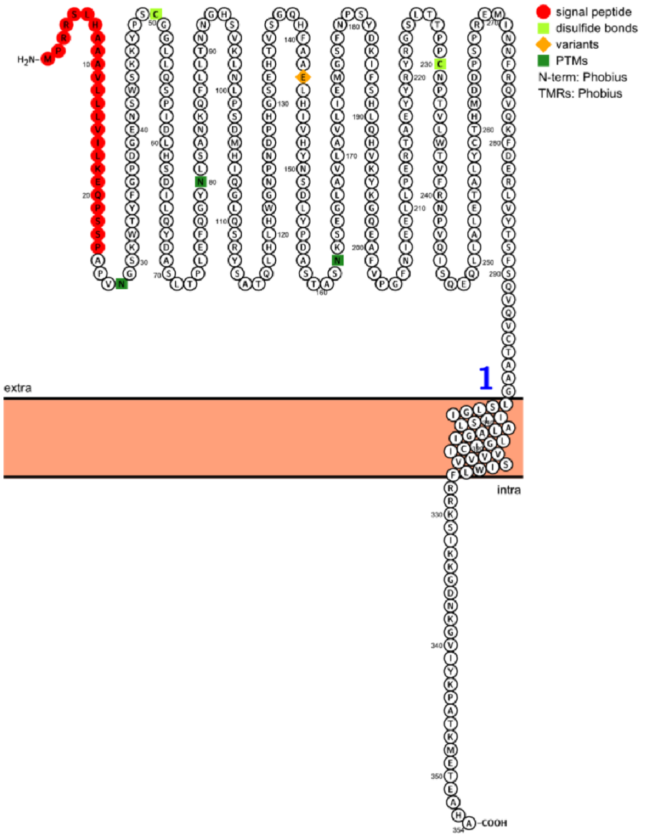

Supplementary Figure 1: CAXII topology. The topology of CAXII was obtained with Protter software (Omasits U, Ahrens CH, Müller S, Wollscheid B. Protter: interactive protein feature visualization and integration with experimental proteomic data. *Bioinformatics* 2014; **30**: 884–886; http://wlab.ethz.ch/protter).

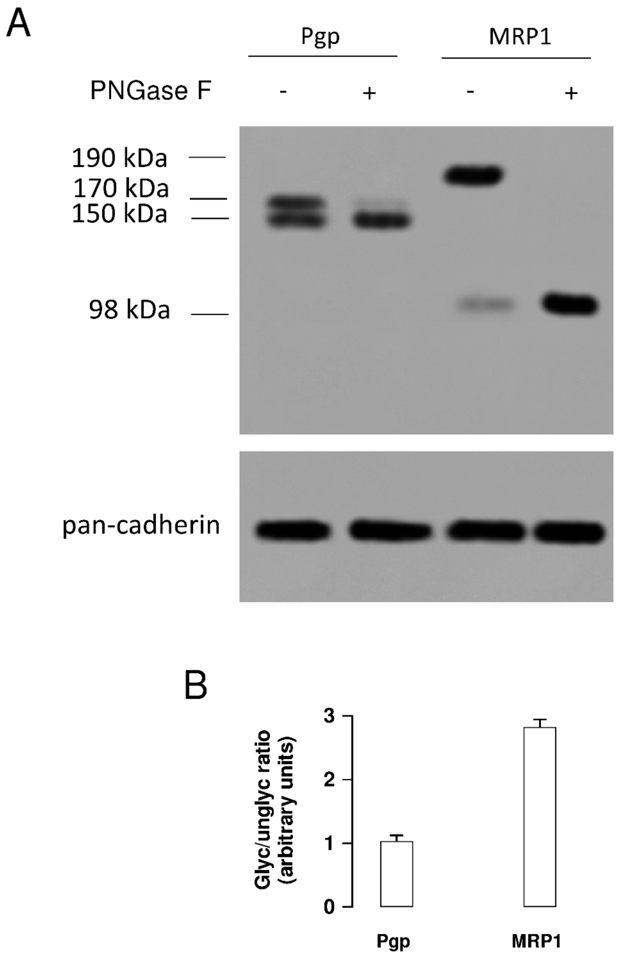

Supplementary Figure 2: Glycosylated versus deglycosylated Pgp and MRP1 in chemoresistant colon cancer cells. **(A)** Biotinylated plasma membrane extracts from human chemoresistant colon cancer HT29/dx cells were analyzed by Western blotting for the expression of Pgp and MRP1, with antibodies recognizing the glycosylated (i.e. 170 kDa band for Pgp, 190 kDa band for MRP1) and the deglycosylated (i.e. 150 kDa band for Pgp, 98 kDa band for MRP1) form of each protein. As internal control, extracts were incubated 1 h at 37°C with 1 μU of recombinant peptide-N-glycosidase F (PNGase F), to remove N-glycosylation. The pan-cadherin expression was used as a control of equal protein loading. The figure is representative of three experiments with similar results. **(B)** The densitometric analysis between glycosylated and deglycosylated bands (glyc/deglyc ratio) was performed with the ImageJ software (http://imagej.nih.gov/ij/).

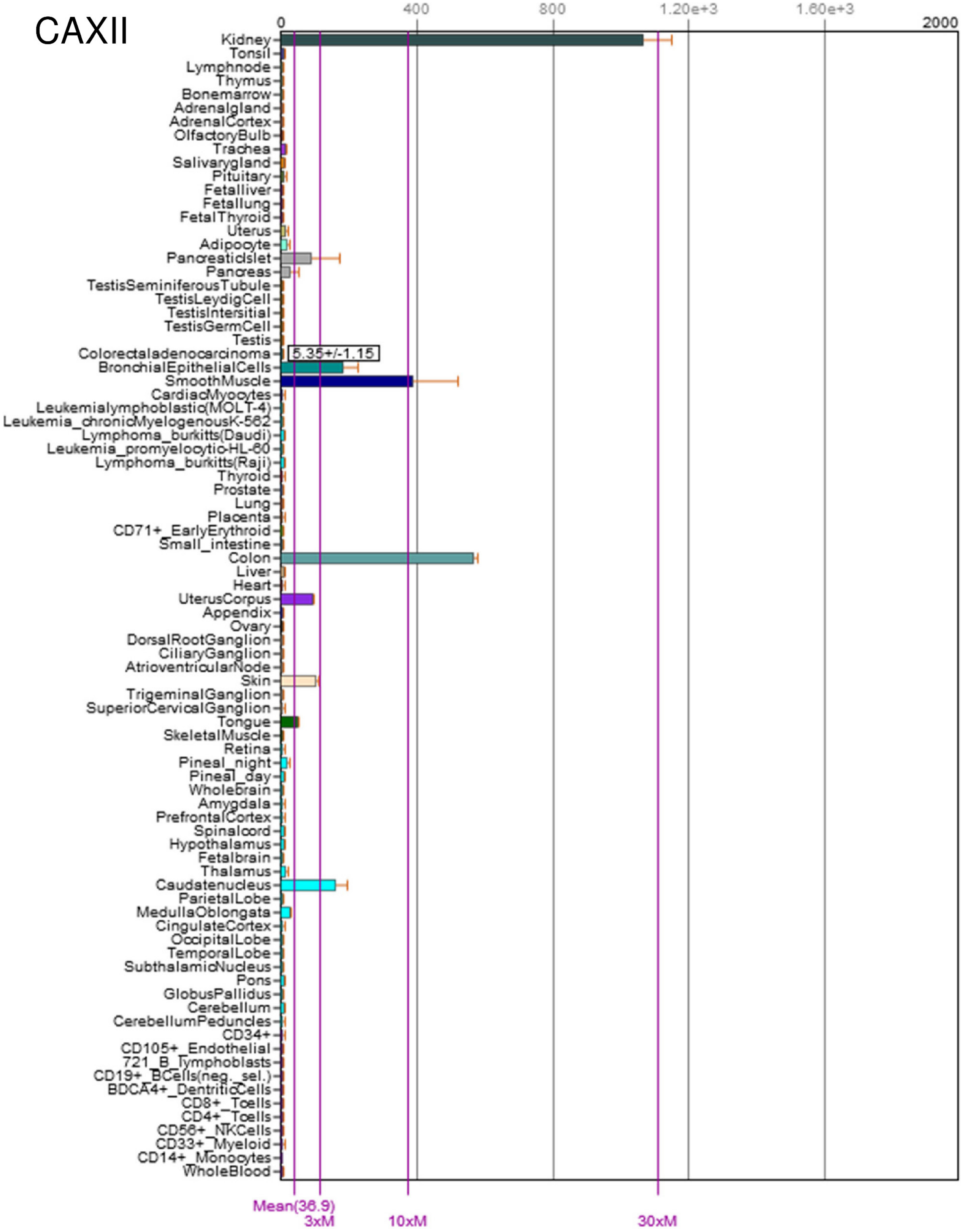

Supplementary Figure 3: Expression of CAXII in different human tissues. The expression level of CAXII was analyzed with the BioGPS tool (http://biogps.org, http://genomebiology.com/2009/10/11/R130), GeneAtlas U133A (http://www.ncbi.nlm.nih.gov/pubmed/15075390), gcrma, probe 203963_at. The relative expression level of CAXII in colon adenocarcinoma is indicated.

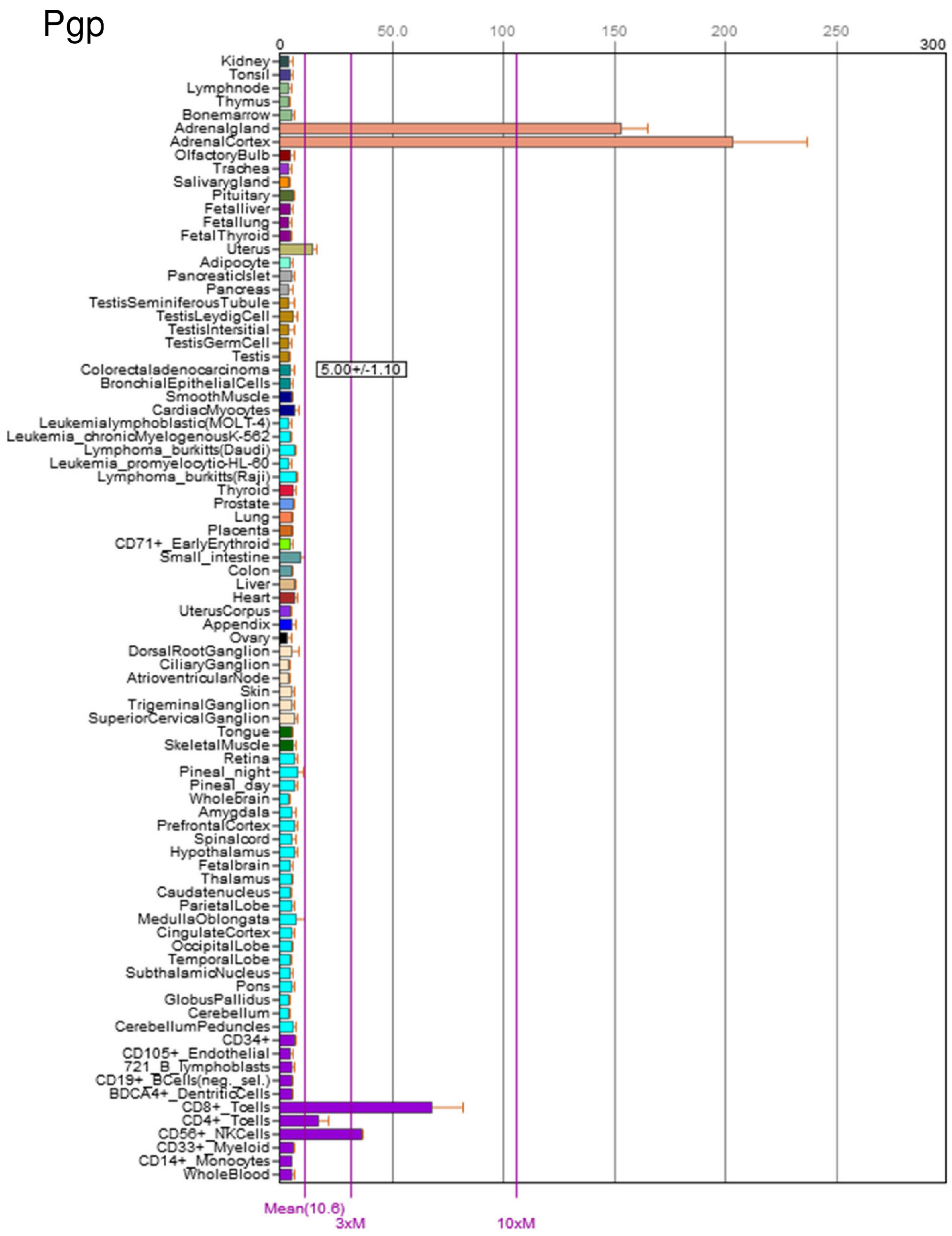

Supplementary Figure 4: Expression of Pgp in different human tissues. The expression level of Pgp was analyzed with the BioGPS tool (http://biogps.org, http://genomebiology.com/2009/10/11/R130), GeneAtlas U133A (http://www.ncbi.nlm.nih.gov/pubmed/15075390), gcrma, probe 209993_at. The relative expression level of Pgp in colon adenocarcinoma is indicated.

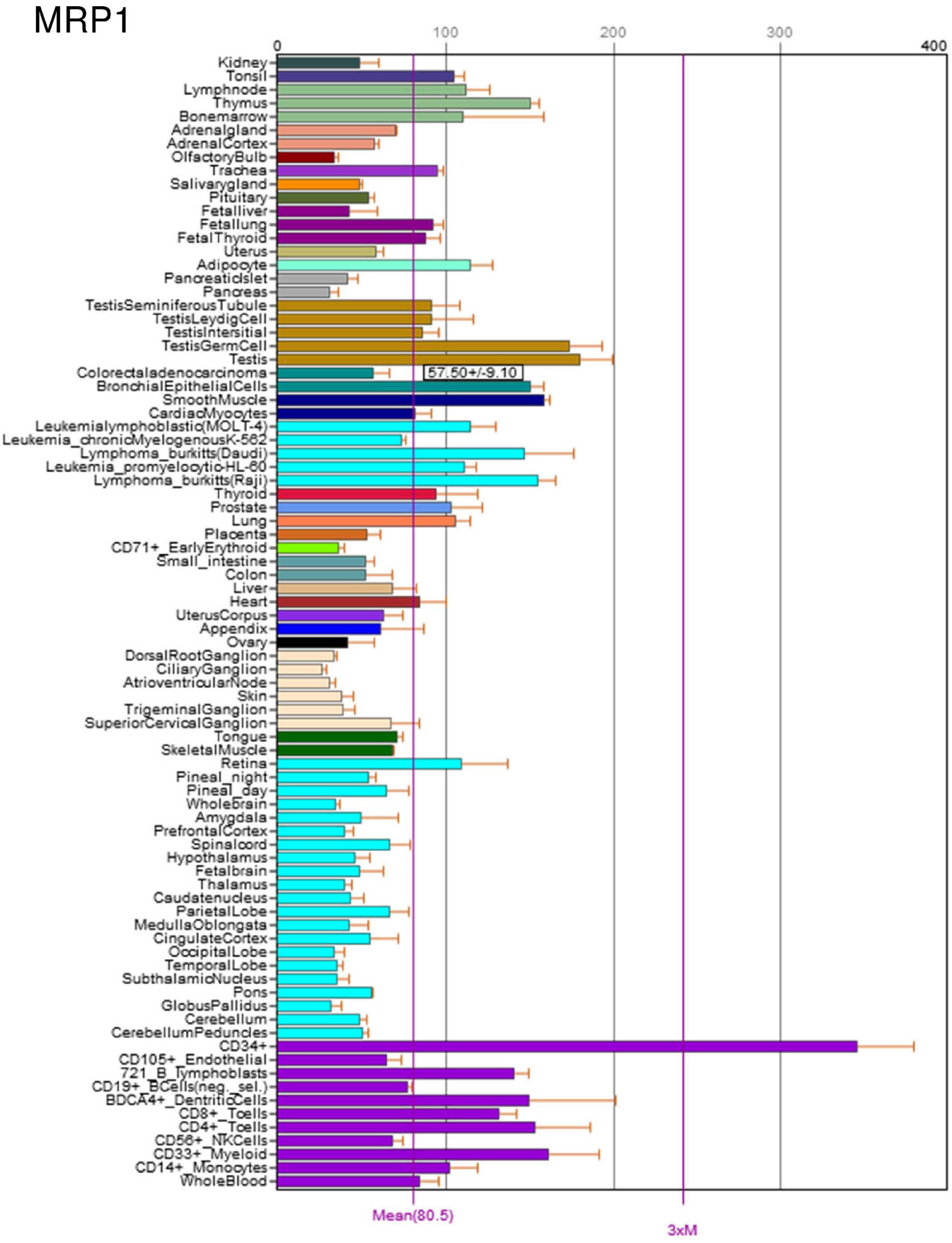

Supplementary Figure 5: Expression of MRP1 in different human tissues. The expression level of MRP1 was analyzed with the BioGPS tool (http://biogps.org, http://genomebiology.com/2009/10/11/R130), GeneAtlas U133A (http://www.ncbi.nlm.nih.gov/pubmed/15075390), gcrma, probe 202804_at. The relative expression level of MRP1 in colon adenocarcinoma is indicated.

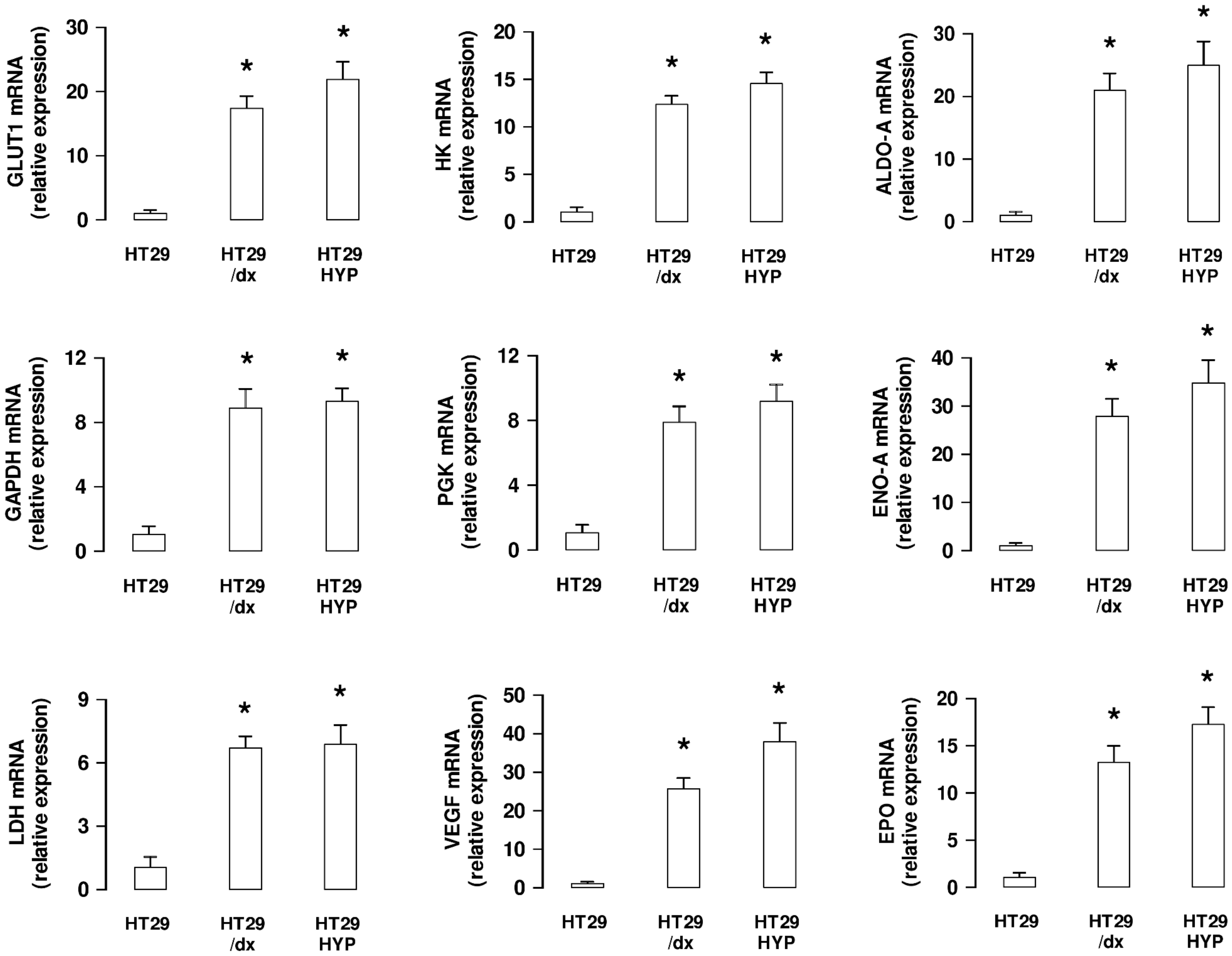

Supplementary Figure 6: Expression of HIF-1α target genes in chemosensitive and chemoresistant colon cancer cells. The mRNA level of glucose transporter 1 (*GLUT1*), hexokinase (*HK*), aldolase-A (*ALDO-A*), glyceraldehyde 3-phosphate dehydrogenase (*GAPDH*), phosphoglycerate kinase (*PGK*), enolase-A (*ENO-A*), lactate dehydrogenase (*LDH*), vascular endothelial growth factor (*VEGF*), erythropoietin (*EPO*) was detected by qRT-PCR in normoxic HT29 and HT29/dx cells. As positive control of HIF-1α activation, the expression of the same mRNAs was measured in HT29 cultured in hypoxic conditions (2% O_2 for 24 h; HYP). Data are presented as means ± SD ($n = 4$). For all panels, versus normoxic HT29: * $p < 0.001$.

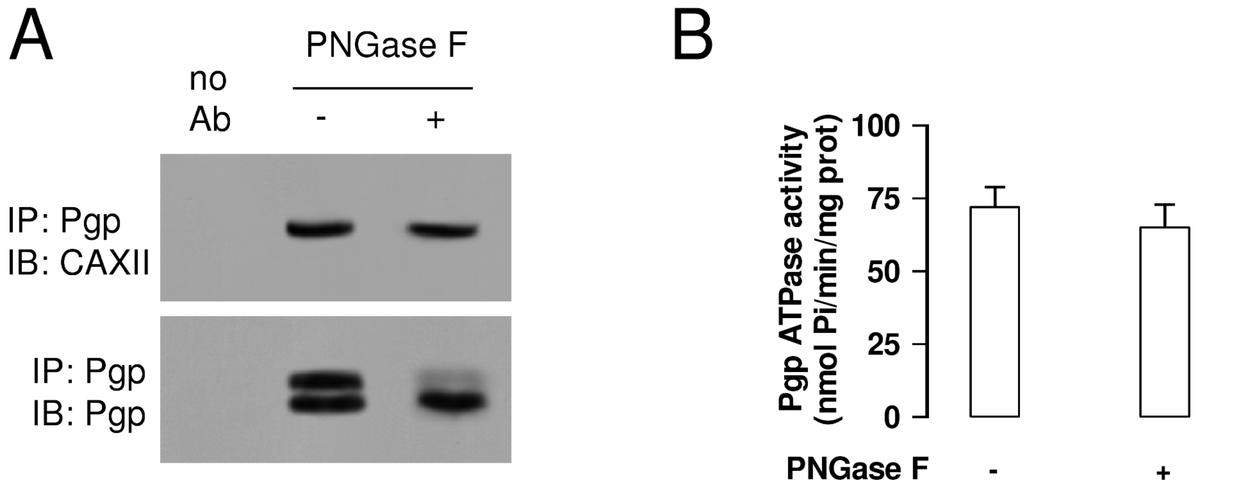

Supplementary Figure 7: CAXII is associated with both glycosylated and deglycosylated Pgp. (A) Biotinylated plasma membrane extracts from human chemoresistant colon cancer HT29/dx cells were incubated 1 h at 37°C with (+) or without (−) 1 μU of PNGase F, immunoprecipitated (IP) with an anti-Pgp antibody recognizing both the glycosylated and the deglycosylated form of Pgp, then immunoblotted (IB) with an anti-CAXII or an anti-Pgp antibody. No Ab: samples immunoprecipitated without antibody. The figure is representative of two experiments with similar results. **(B)** Pgp ATPase activity was measured spectrophotometrically on Pgp-rich vesicles extracted from membrane fractions, pre-treated or not with PNGase F as reported in A. Data are presented as means ± SD (*n* = 3).

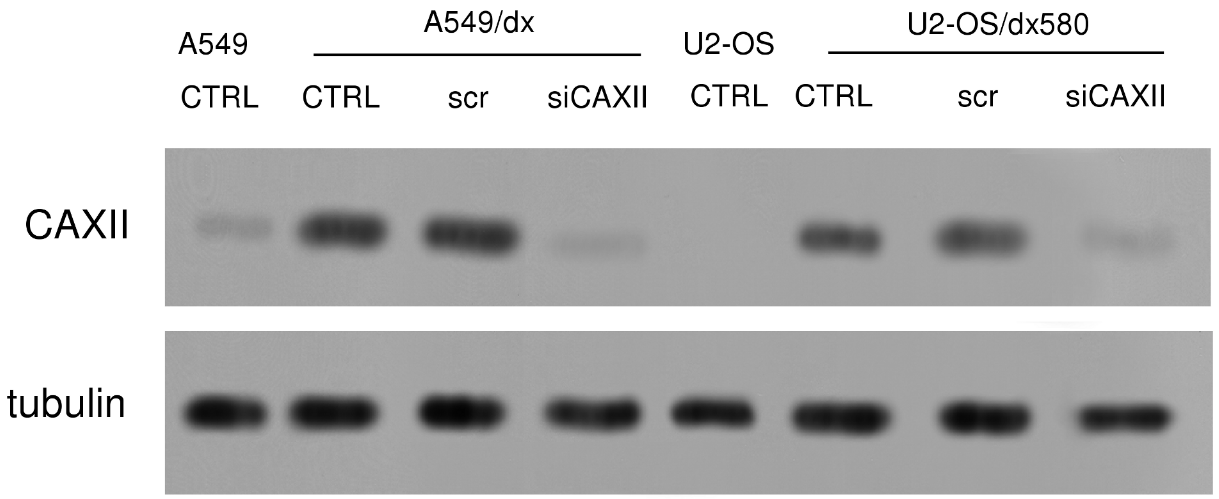

Supplementary Figure 8: CAXII silencing in lung and osteosarcoma chemoresistant cells. Human chemoresistant lung cancer A549/dx cells and osteosarcoma U2-OS/dx580 cells were cultured for 48 h with fresh medium (CTRL), treated with a non targeting scrambled siRNA (scr) or with a CAXII-targeting specific siRNA pool (siCAXII). Chemosensitive A549 and U2-OS cells were included as control. The expression of CAXII was measured in whole cell lysates by Western blotting. The β-tubulin expression was used as a control of equal protein loading. The figure is representative of two experiments with similar results.

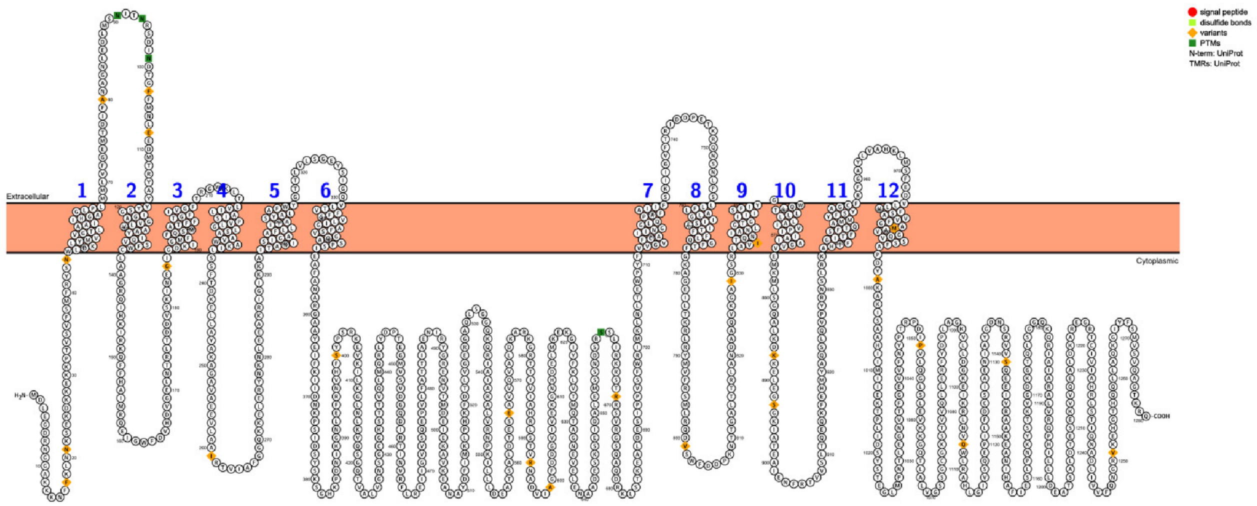

Supplementary Figure 9: Pgp topology. The topology of Pgp was obtained with Protter software (Omasits U, Ahrens CH, Müller S, Wollscheid B. Protter: interactive protein feature visualization and integration with experimental proteomic data. *Bioinformatics* 2014; **30**: 884–886; http://wlab.ethz.ch/protter).

Supplementary Table 1: Quantitative analysis of glycoproteins present on HT29 and HT29-dx cells surface

Supplementary Table 2: Primers sequence for qRT-PCR

Gene	Forward primer	Reverse primer
CAXII	CCACACGACGGGTACTTCTT	AAAGGAACAGCCTTCCAGC
Pgp	ATTCCTCGAGAAACTGCGAA	CACTTCAGGAAGCAACCAG
HIF-1α	TGGCTGCATCTCGAGACTTT	GAAGACATCGCGGGGAC
GLUT1	CCTGCAGTTTGGCTACAACA	TAACGAAAAGGCCCACAGAG
HK	AGACGCACCCACAGTATTCC	CGCATCCTCTTCTTCACCTC
ALDO-A	GCTATGGCCTTTTCCTTTCC	ATGCTCCCAGTGGACTCATC
GAPDH	GAAGGTGAAGGTCGGAGT	CATGGTGGAATCATATTGGAA
PGK	TCTCATGGATGAGGTGGTGA	CTTCCAGGAGCTCCAAACTG
ENO-A	GCTCCGGGACAATGATAAGA	TCCATCCATCTCGATCATCA
LDH	TGGGAGTTCACCCATTAAGC	AGCACTCTCAACCACCTGCT
VEGF	ATCTTCAAGCCATCCTGTGTGC	GCTCACCGCCTCGGCTTGT
EPO	CAGACTTCTACGGCCTGCTG	GCTGAACACTGCAGCTTGAA
CAIX	GTCTCGCTTGGAAGAAATCG	AGAGGGTGTGGAGCTGCTTA
S14	CGAGGCTGATGACCTGTTCT	GCCCTCTCCCACTCTCTCTT
